# When Can Making a Drawing Hinder Problem Solving? Effect of the Drawing Strategy on Linear Overgeneralizations and Problem Solving

**DOI:** 10.3389/fpsyg.2020.00506

**Published:** 2020-03-27

**Authors:** Janina Krawitz, Stanislaw Schukajlow

**Affiliations:** Department of Mathematics, University of Münster, Münster, Germany

**Keywords:** drawing strategy, geometry problems, illusion of linearity, linear overgeneralizations, monitoring, problem solving, self-generated drawing

## Abstract

The strategy of making a drawing has been claimed to facilitate mathematical problem solving. However, De Bock et al. (2003) surprisingly found that drawing negatively affected performance in solving non-linear geometry problems, in which the area or volume of similar figures or solids had to be determined by a given scaling factor. The authors suggested that making a drawing increased the number of overgeneralizations and negatively affected students’ performance. Our study involves a partial replication and also an important validation and extension of this study by addressing two factors: low-quality drawing strategy and poor visual monitoring, both of which might explain the negative effect of drawing. First, we expected that improving the quality of the drawing strategy by prompting students to highlight important information in their drawings would diminish the negative effect of the drawing strategy. Second, we expected that fostering visual monitoring while drawing, by offering problems with small scaling factors, would diminish the negative effect of the drawing strategy. We conducted a randomized controlled trial with 180 students (ninth- to eleventh-graders) to investigate the effects of drawing and visual monitoring on solving non-linear geometry problems. Our results replicated the previous finding that drawing negatively affects performance. We confirmed that linear overgeneralizations are a prevalent reason for this finding. Elaborating on previous findings revealed that the quality of the drawing strategy but not visual monitoring was responsible for the effect of the drawing strategy on linear overgeneralizations. Furthermore, an exploratory analysis of students’ awareness of linear overgeneralizations indicated that improving the quality of drawing strategy and enhancing visual monitoring did not lead to a greater awareness of the mistakes learners made because of linear overgeneralizations. We conclude that the way the drawing strategy is used determines whether it is useful or damaging, and more efforts are essential to enable students to apply it appropriately.

## Introduction

Making a drawing is considered a powerful strategy in mathematical problem solving (Pólya, 1945). According to the theory of external representations (Cox, 1999), it can support problem solving by helping problem solvers organize the information, and it can make missing and implicit information (e.g., relations between objects) explicit. Therefore, it deepens understanding and facilitates self-explanatory activities. Empirical evidence for the benefits of drawing for problem solving has been found in various studies (e.g., Van Essen and Hamaker, 1990; Hembree, 1992; Zahner and Corter, 2010; Rellensmann et al., 2016). However, the drawing strategy does not seem to be helpful for solving some types of problems, and surprisingly, it can even be disadvantageous by decreasing students’ performance in solving non-linear geometry problems (De Bock et al., 2003). It seems that drawing leads to an increase in students’ well-known tendency to engage in linear overgeneralizations, which means that learners tend to apply linear models to non-linear situations (Van Dooren et al., 2005). From a broader perspective, this finding demonstrates the need to investigate the processes elicited by the drawing strategy and the key factors that determine the beneficial use of the strategy. On the basis of these considerations, the goals of the present study are twofold: (a) to replicate De Bock et al. (2003) surprising finding that drawing hinders students’ ability to solve non-linear geometry problems and (b) to find explanations for this unexpected phenomenon. On the basis of prior research, we suggest that the insufficient quality of the drawing strategy and a lack of opportunity to use the drawing strategy for monitoring purposes are crucial factors that have contributed to the negative effects of drawing. Our aim is to clarify whether these factors come into play while students solve non-linear geometry problems and, more specifically, whether it is possible to diminish the negative effect of drawing by addressing these factors.

## Drawing Strategy and Linear Overgeneralizations

### Self-Generated Drawing

External visual representations are omnipresent in contexts of learning and education. Thus, they serve different functions. First, the ability to deal with external visual representations such as drawings can be considered a learning goal on its own because in many situations in class and everyday life, it is necessary to interpret, construct, and work with them (National Governors Association Center for Best Practices and Council of Chief State School Officers, 2010). Second, they have been claimed to enhance learning by relieving working memory, promoting self-explanation activities, and leading to a deeper understanding of the learning material (Cox, 1999; Mayer, 2005; Van Meter and Garner, 2005). An important distinction has to be made between ready-made and self-generated external visual representations. For the latter, learners construct representations on their own, which means that they are actively involved in externalizing their mental representation, which includes the processes of organizing, selecting, and integrating the information given in the problem (Van Meter and Garner, 2005). In the present paper, we focus on self-generated drawing. We define the drawing strategy as the process of constructing an external visual representation that corresponds to the mathematical problem structure and is aimed at solving the problem (Van Meter and Garner, 2005).

Self-generated drawing influences the process of problem solving, as it guides the learner’s attention and directs or even determines his or her actions. Theories of cognition assume that when beginning to solve a problem, humans construct an internal representation of the problem situation called a mental model (Johnson-Laird, 1980). While drawing, the mental model is transformed into an external visual representation (i.e., a drawing). This process is more than a simple translation because it involves a re-organization of the given information and dynamic iterations between the mental model and the externalized model in order to match both representations (Cox, 1999). Re-organizing the information can make key elements of the problem and its relations visible so that the information can be more easily processed after a drawing is constructed (see section “Quality of Drawing Strategy”) (Larkin and Simon, 1987). In order to successfully solve the problem, it is crucial that the structure of the problem be adequately presented in the external visual representation. Otherwise, drawing could cause perceptual and cognitive biases, which might guide the problem solver away from the goal (Zhang, 1997; Cox, 1999).

Studies investigating the effect of drawing on problem solving performance have arrived at divergent findings. A number of empirical studies have found that drawing positively affects problem solving in mathematics (Van Essen and Hamaker, 1990; Hembree, 1992; Zahner and Corter, 2010; Rellensmann et al., 2016). Strong support for the benefits of the drawing strategy were provided by the meta-analysis conducted by Hembree (1992). Training students to draw was identified as the most effective treatment for improving problem solving performance compared with training them to use other strategies such as handling extraneous data, verbalizing concepts, or using guess-and-test procedures. However, several factors seem to determine whether the drawing strategy is helpful or not. For example, Van Essen and Hamaker (1990) found that drawing showed a positive effect for fifth-graders but not for first- and second-graders, indicating that the benefits of making a drawing depend on the specific difficulties learners encounter while solving problems. Another important factor seems to be the type of problem because, for some types of problems, drawing was shown to be beneficial [e.g., probability problems (Zahner and Corter, 2010) or arithmetic word problems (Van Essen and Hamaker, 1990)], whereas for other types of problems, in particular non-linear geometry problems, no effect (De Bock et al., 1998) or even a negative effect (De Bock et al., 2003) was found. The most important factor that determined whether making a drawing was beneficial or not seemed to be the quality of the drawing strategy, which we address in the next section.

### Quality of Drawing Strategy

The quality of the drawing strategy refers to the correctness and the explicit presentation of key information. Accordingly, the high-quality use of the drawing strategy means that the drawing as the product of the drawing process is correct and complete with regard to the important elements and their relations. Both criteria need to be met so that the rapid processing capabilities of a learner’s visual system can be used to make perceptual judgments instead of depending on difficult logical inferences (Cox, 1999).

Research on self-generated drawing has shown that the benefits of drawing are strongly related to the quality with which the drawing strategy is applied (Van Garderen and Montague, 2003; Uesaka et al., 2007; Schwamborn et al., 2010; Mason et al., 2013; Rellensmann et al., 2016). Learners who apply the drawing strategy in a high-quality way perform better on problem solving and learning outcome tests than learners who apply the drawing strategy in a lower quality manner. Problem solving research has shown that students often fail to use a high-quality drawing strategy because they tend to generate pictorial representations with merely a decorative function instead of depicting important elements and their relations (Hegarty and Kozhevnikov, 1999; Van Garderen and Montague, 2003). For non-linear geometry problems, a qualitative analysis of students’ solutions indicated that the quality with which the drawing strategy was applied was usually too poor – regarding correctness and the explicit presentation of key information – to help students solve the problems (De Bock et al., 1998). Hence, the request to draw is probably not enough, and it might be necessary to give students support that will render the drawing strategy more helpful for problem solving. Empirical indications for this claim have been provided in studies of text-based learning. In the study by Van Meter (2001), applying the drawing strategy was more effective for conditions in which students’ drawing process was supported by providing illustrations or prompts to compare the illustrations with self-generated drawings. It was found that supporting students’ drawing activities had a positive effect on the performance of comprehensive free recall but not recognition items. These results indicate that improving the quality of the drawing strategy is essential for students’ performance if the assessment requires them to build connections between the information given in the problem, as is the case when students solve non-routine mathematical problems.

### Visual Monitoring

Another important factor in the context of research on self-generated drawing is that the drawing strategy can enhance monitoring processes. Monitoring has been claimed to be essential for problem solving (Flavell, 1979) and plays an important role in detecting incorrect intuitions and misconceptions such as linear overgeneralizations (Van Dooren et al., 2004). The drawing strategy can be considered a tool that can be used for monitoring for the following reasons. When students use the drawing strategy, they construct a visual representation on the basis of an abstract symbolic representation. As visual representations are limited in abstraction, they aid processability and lead to the generation of new information (Stenning and Oberlander, 1995). Hence, the drawing strategy can be used to detect inconsistencies. In particular, in problem solving, the drawing strategy can be applied with the goal of revealing mistakes and inaccuracies in the student’s mental model of the problem situation. In the following, when the drawing strategy is applied for monitoring purposes, we refer to this as visual monitoring.

Empirical evidence for the claim that drawing strategy can be used for monitoring purposes can be derived from the study by Stylianou (2011). The problem solving activities of experts (mathematicians) and novices (middle school students) were analyzed by using qualitative methods in order to identify the purposes of the drawing strategy. Both experts and novices used the drawing strategy to monitor the progress of problem solving, including checking the correctness and making informed decisions about subsequent actions. However, in contrast to experts, middle school students engaged in visual monitoring only infrequently and – if at all – to verify their result at the end of the problem solving process. This finding indicates the importance of supporting school students in their visual monitoring activities.

Further indications come from text-based learning research. Van Meter (2001) analyzed the think-aloud protocols of fifth- and sixth-graders who read a science text under two conditions: Self-generated drawing compared with working with ready-made drawings. It was found that students who used self-generated drawing engaged in significantly more monitoring events, such as looking back and self-questioning, compared with students who worked with ready-made drawings. Further, monitoring events were higher when students received additional support during their drawing activity. Hence, supporting students’ drawing activities is crucial for determining the way in which the drawing strategy is used. In sum, drawing seems to fulfill monitoring purposes, and supporting the drawing activities increases visual monitoring. However, research has yet to determine the extent to which these results are valid for mathematical problem solving.

### Linear Overgeneralizations

Misconceptions often emerge when learners generalize their prior knowledge by systematically activating it in contexts in which it is inappropriate (Smith et al., 1993). A well-known example of such a misconception is the “illusion of linearity,” the tendency to apply linear models to non-linear situations, which will be referred to as linear overgeneralizations in the following. Linearity and especially proportionality can be considered the simplest but also the most important property of mathematical relationships (two quantities change with an equal amount of growth). Many facts of the real world are based on linear and proportional relationships. Also in mathematics education, linearity plays a central role and emerges during the time children are in school in the contexts of different mathematical topics ranging from arithmetic word problems, to linear functions, to advanced concepts such as the diameter and circumference of a circle. However, the intensive treatment of linearity might result in the disadvantage that some students will develop false conceptions, namely, the idea that linear models have a kind of universal validity. As a consequence, they might mistakenly transfer the principle of linearity to non-linear contexts.

Empirically, students’ strong tendency to engage in linear overgeneralizations has been supported by a large amount of research that has included different age groups ranging from primary school (Van Dooren et al., 2005) to university students (Esteley et al., 2010) and has referred to different mathematical domains such as arithmetic word problems (Van Dooren et al., 2005, 2010), algebraic patterns (Stacey, 1989), geometry (De Bock et al., 1998, 2003; Ayan and Bostan, 2018), and probability (Van Dooren et al., 2003). More specifically, linear overgeneralizations seemed to increase after linear problems were taught in class (Van Dooren et al., 2005), supporting the assumption that students’ experiences with linear concepts in the mathematics classroom are responsible for their strong tendency to engage in linear overgeneralizations. However, even very young students (second- and third-graders) tend to give linear answers to non-linear problems, indicating that other factors also need to be taken into account. One of these factors could be individuals’ tendency to reduce information in their environment into structures that are as simple as possible, which is known as the “Law of simplicity” (Chater and Vitányi, 2003). As linearity and in particular proportionality is the simplest form of relationship between two quantities, this bias may also occur independent of the effect of students’ experiences with linear problems in class.

One of the most investigated types of problems with regard to linear overgeneralizations is the non-linear geometry problem, in which students are asked about how enlarging or reducing a geometrical figure affects its area or volume. For example: “You need approximately 400 g of flower seed to lay out a circular flower bed with a diameter of 10 m. How many grams of flower seed would you need to lay out a circular flower bed with a diameter of 20 m?” (De Bock et al., 1998, p. 68). A series of studies demonstrated that students between the ages of 12 and 16 were usually not able to solve such problems (De Bock et al., 1998, 2002b, 2003). Overall, these studies reported particularly low solution rates for younger students (rates of 2% and 7% for correct solutions for 12- to 13-year-olds), but wrong answers were usually given among the older students too (23% correct solutions for 13- to 14-year-olds; 17%, 22%, 43% correct responses for 15- to 16-year-olds). Building on these findings, De Bock et al. (2002a) conducted an interview study to investigate which aspects are responsible for the frequent appearance of linear overgeneralizations. They found that some of the students had the firm conviction that any relationship between two variables could be expressed by a constant of proportionality. However, the majority of the students used linear models in an intuitive manner, without being aware of the model they chose. Students apparently do not recognize the mistakes they make on the basis of linear overgeneralizations and therefore probably perceive that their solutions to these problems are correct even when they are incorrect.

Further, a teaching experiment conducted by Van Dooren et al. (2004) showed that it is possible to decrease the number of linear overgeneralizations in the solutions to such problems. In 10 experimental lessons, major holes in students’ prior geometrical knowledge and their linearity preconceptions were addressed by eliciting cognitive conflicts. Further aims of the intervention were to facilitate students’ meta-conceptual awareness, including monitoring and enhancing a deeper understanding from the use of multiple external representations of the central mathematical contents. Although the automatic use of linear strategies was successfully reduced, some of the students in the experimental group still tended to engage in linear overgeneralizations, whereas others started to also apply non-proportional strategies to proportional problems, indicating that the intervention was not successful in terms of fostering a deep conceptual understanding of differences in linearity and non-linearity in some of the students. These results provide the first hints that external representations might be beneficial for diminishing linear overgeneralizations. Further support comes from the study by De Bock et al. (2002b) who found that providing ready-made drawings of the original and scaled figures on graph paper had a positive albeit small effect on solution rates for non-linear geometry problems. Graph paper allows comparisons to be made of the areas of the figures by counting the squares and thus facilitates the recognition of the non-linear relationship of the areas.

We view these findings as initial indications of the importance of external representations for overcoming linear overgeneralizations and performance. Further indications pointing in the opposite direction come from research on self-generated drawing.

### Effects of the Drawing Strategy on Linear Overgeneralizations and Performance

A series of experimental studies investigated the impacts of making a drawing on linear overgeneralizations and performance. In one of these studies (De Bock et al., 1998), students in a drawing condition were instructed to draw before solving each item. The instructions were given at the beginning of the test using an example item. Contrary to theoretical considerations, no effect of making a drawing on performance was found. The percentage of correct solutions for the group of 12- to 13-year-old students remained at only 2% and was also found to be low for 15- to 16-year-old students regardless of the drawing instructions.

Different drawing instructions were implemented in a subsequent study (De Bock et al., 2003). In the drawing condition, students between the ages of 13 and 16 were given a drawing of a geometrical object for each problem (e.g., a square) and were asked to complete the drawing by supplementing a scaled copy of the object using the given scaling factor. The surprising finding was that students who received these instructions showed significantly lower solution rates than the control group (23% vs. 44%). An additional analysis of the solution processes from this study suggested that self-generated drawing did not elicit visual solution strategies such as “paving” – determining the area of a plane figure by paving it with similar Figures – and hence, the drawing strategy was apparently not applied appropriately. This is a potential reason why drawing is not beneficial, but it does not explain the negative effect. An analysis of the problems used in this study provided another reason for this result. Making a drawing might hinder students’ progress while solving non-linear problems because the process of drawing might divert their attention to unimportant elements or even to elements that could interfere with their solution process: Figures are typically depicted by their circumferences, which change linearly through scaling. In the process of drawing, learners work with linear relationships and may erroneously transfer them to the area. This might render the quality of the drawing strategy insufficient because key information (i.e., the area) is not made salient in the drawing. Increasing the quality of the drawing strategy by highlighting the area in the drawings may guide learners’ attention to the important elements of the problem, thus helping them identify non-linear properties while drawing.

Another aspect that also affects the recognition of non-linearity concerns visual monitoring. Visual monitoring should reveal that the area changes non-linearly through scaling. However, visual monitoring might potentially not come into effect if the size of the scaling factor is too large. For problems with small scaling factors (e.g., doubling the side length), the difference between the area or volume of the original and of the modified figure becomes salient while drawing so that visual monitoring should uncover the non-linear relationship. Whereas for large scaling factors (e.g., if the side length is twelve or more times larger), the difference in the area or volume cannot be easily visually estimated. Consequently, it can be expected that visual monitoring, enabled by using small scaling factors, can help learners overcome their difficulties with linear overgeneralizations so that they will demonstrate better performance in problem solving. However, even if students recognize the non-linear relationship by engaging in high-quality drawing or visual monitoring, they are not necessarily able to solve the problem. Instead, they might change the problem by imposing an inappropriate structure that enables them to apply available solution strategies (Goos, 2002). It is possible that they might detect the non-linear property of the area but nevertheless use linear models to solve the problem because they lack adequate solution strategies (Weber, 2001). Students who recognize the non-linear relationship of the areas are probably aware of their inappropriate application of linear overgeneralizations and will consequently perceive their lower performance in solving the problems than students who do not recognize the non-linear relationship. Thus, we assume that students’ perceptions of their performance in solving non-linear geometry problems might be an indicator of students’ awareness of the non-linear property of the problems. Data on students’ perceived performance will help us interpret the effect of drawing quality and visual monitoring on linear overgeneralizations and performance.

## Research Questions and Expectations

On the basis of theoretical considerations and prior empirical findings, we posed the following research questions:

1. Does the instruction to make a drawing of the scaled figure lead to a larger number of linear overgeneralizations and have a negative effect on problem solving performance of non-linear geometry problems?
2. Does improving the quality of the drawing strategy by highlighting important information in the drawing decrease the number of linear overgeneralizations and diminish the negative effect of the drawing strategy on problem solving performance?
3. Does visual monitoring decrease the number of linear overgeneralizations and diminish the negative effect of the drawing strategy on problem solving performance?
4. Does drawing or visual monitoring affect students’ perceived performance when solving non-linear geometry problems?

### Expectations for Research Question 1 (Drawing)

The first research question addresses the replication of De Bock et al.’s (2003) finding that making a drawing hinders students’ ability to solve non-linear geometry problems. Following theoretical domain-specific considerations regarding the reasons for learners’ linear overgeneralizations, we assume that self-generated drawing distracts learners and draws their attention toward elements of the problem that interfere with their solution process, for example, the linear relationship of the circumferences of the original and scaled figures in problems with rectilinear plane figures. Because of the very common tendency to engage in linear overgeneralizations (Van Dooren et al., 2008), they may erroneously transfer the linear relationship of the circumferences to the non-linear relationship of the areas. The same considerations can be made for problems with non-rectilinear figures and solids regarding the linear property of the diameter and the non-linear property of the volume. Thus, we expected the drawing strategy to increase the number of linear overgeneralizations and negatively affect problem solving performance.

### Expectations for Research Question 2 (High-Quality Drawing):

We expected that increasing the quality of the drawing strategy by highlighting key information would diminish the negative effect of the drawing strategy. Hence, we expected that students applying a high-quality drawing strategy and students not applying the drawing strategy would show the same number of linear overgeneralizations and performance in solving non-linear geometry problems. Further, we expected fewer linear overgeneralizations and higher performance from students who applied the high-quality drawing strategy than students who used the lower quality drawing strategy. The rationale behind these expectations is that the effects of the drawing strategy strongly depend on drawing quality. One key characteristic of a high-quality drawing is the explicit presentation of key information. For non-linear geometry problems, mistakes are made due to an inappropriate focus on the side length or the diameter of the figure or solid and its linear properties instead of considering the area or the volume, respectively. Hence, highlighting the area or volume of the drawn figure or solid will improve the quality of the drawing strategy and should lead to fewer linear overgeneralizations and a higher performance than the use of a lower quality drawing strategy.

### Expectations for Research Question 3 (Visual Monitoring)

We expected that visual monitoring would diminish the negative effect of the drawing strategy. Consequently, visual monitoring while drawing should lead to a similar number of linear overgeneralizations and a similar performance in solving non-linear geometry problems in comparison with solving the problems without a drawing. In addition, we expected that visual monitoring would lead to fewer linear overgeneralizations and a higher performance than drawing without visual monitoring. We enhanced visual monitoring by using small scaling factors instead of large ones on the basis of our assumption that for small scaling factors, the non-linear relationship of the areas becomes salient while drawing. Consequently, visual monitoring could help overcome the linear overgeneralizations that were elicited by the drawing strategy.

### Expectations for Research Question 4 (Effects on Perceived Performance)

The fourth research question followed an exploratory approach. Thus, we did not have specific expectations. The aim of analyzing students’ perceived performance is to increase the validity by using different indications of students’ success (Rovers et al., 2019) and to gather further information that helps to explain the findings from our experimental study. Students’ perceived performance in the drawing and visual monitoring conditions will provide indications of students’ awareness of the non-linear property of the problems. Students who notice the non-linear relationship because they make a high-quality drawing or engage in visual monitoring might lack the mathematical knowledge to proceed and will therefore nevertheless stick to the application of linear models and will report lower perceived performance.

## Materials and Methods

### Sample and Procedure

The present study involved 198 students (57.1% female, mean age = 16.15 years) from nine classes, including ninth-graders (12.6%), tenth-graders (48.5%), and eleventh-graders (38.9%). Students came from four high-track schools (German Gymnasium) and one comprehensive school (German Gesamtschule).

**FIGURE 1:**
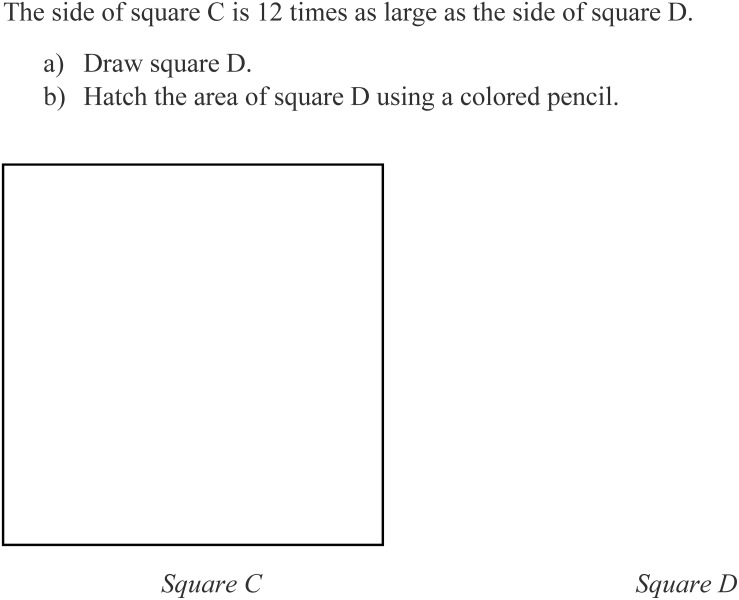
Sample item with drawing and highlighting instructions. Tasks were adopted from De Bock et al. (2003, p. 449).

Students in each class were randomly assigned to one of five groups: Students in the experimental conditions received either drawing (D) or drawing with highlighting (DQ) instructions, aimed at increasing the quality of the drawing strategy, and the test version with either large [11, 12, or 13 as used in the study by De Bock et al. (2003)] or small scaling factors (3, 4, or 5), aimed in enhancing visual monitoring (V− and V+ groups). These conditions resulted in four combinations of experimental conditions (DV−, DV+, DQV−, DQV+). Students in the control group (CG) received no drawing instructions and the test version with large scaling factors as in the study by De Bock et al. (2003). All groups worked on a paper-and-pencil test consisting of four experimental items, which were non-linear geometry problems, and three additional buffer items. All items were taken from the study by De Bock et al. (2003). Drawing and drawing with highlighting instructions were embedded in each item on the test. Figure 1 shows a sample item with drawing with highlighting instructions as used in the version of the test that was administered in the DQ condition. Students in the D group received the same drawing instructions (part a) but no highlighting instructions (part b).

After taking the test, students completed a questionnaire. The aim of the questionnaire was to collect data on how solving non-linear geometry problems and the experimental treatment were perceived by the students. Thus, the questionnaire included four statements for measuring students’ perceived performance.

### Treatment Check

To check the implementation of the treatment, we examined whether students in the experimental and control groups followed the instructions. The results confirmed that students followed the drawing instructions and the instructions to draw and highlight. As intended, the number of drawings in the D groups was significantly higher than in the CG [96.1% vs. 40.2%; *t*(43.636) = 7.542, *p* < 0.001, *d* = 1.903]. Further, the number of highlighted drawings in the DQ groups was significantly higher than in the CG [80.0% vs. 0.65%; *t*(84.756) = 22.526, *p* < 0.001, *d* = 3.608] and D groups [80.0% vs. 2.4%; *t*(109.960) = 15.798, *p* < 0.001, *d* = 3.033]. However, 19 of 41 participants of the control group made at least for one of the items a spontaneous drawing. These students seem to perform similar or even better than students who did not make a drawing (50.0% vs. 45.5% correct solutions; 18% vs. 29% linear overgeneralizations). To ensure that spontaneous drawings did not distort our results, we again addressed our research questions by analyzing an adjusted subsample. The adjusted subsample included only students who acted in accordance with their condition. As our analysis revealed nearly the same effect sizes for the adjusted subsample and the whole sample, we analyzed the whole sample in our study.

In addition, we examined students’ age and last mathematics grade by computing an ANOVA to ensure the comparability of the treatment conditions. As expected, no significant difference between the groups was found (*p* > 0.10).

### Measures

#### Linear Overgeneralizations and Problem Solving Performance

Linear overgeneralizations were estimated by analyzing whether the solution was based on a linear model (coded 1) or not (coded 0). Students’ performance was analyzed by assigning a score of 1 for the correct solution and a score of 0 for an incorrect solution. Two independent coders were involved in scoring the test booklets. Inter-rater reliability was calculated for each problem on a subset of 20% of the test booklets which were scored by both coders with sufficient inter-rater agreement (Cohen’s κ ≥ 0.773). Reliability was satisfactory (Cronbach’s α = 0.729 for linear overgeneralizations and 0.754 for performance). All problems were taken from De Bock et al. (2003) and are listed here in the version for the V− groups in Table 1.

**TABLE 1:** Experimental items in the V− groups.

Experimental items in the V− groups
The side of square C is 12 times as large as the square D. If the area of square C is 1,440 cm^2^, what’s the area of square D?
The diameter of a circle E is 11 times as large as the diameter of a circle F. If the area of circle E is 242 cm^2^, what’s the area of circle F?
The side of a cube G is 13 times as large as the side of a cube H. If the volume of cube G is 2,197 cm^3^, what’s the volume of cube H?
The diameter of sphere M is 12 times as large as the diameter of a sphere N. If the volume of sphere M is 172,800 mm^3^, what’s the volume of sphere N?

*All items were adapted from De Bock et al. (2003). For students in the V+ groups, the same problems were used but with smaller scaling factors (3, 4, and 5).*

#### Perceived Performance

Students rated the statements on the questionnaire using a five-point Likert scale (from full disagreement to full agreement). The scale for measuring perceived performance was adapted from prior studies (Hänze and Berger, 2007; Schukajlow and Krug, 2014; Schukajlow et al., 2015, 2019a). It included four items: “I noticed that I really understood the arithmetic problems”; “I felt able to master the arithmetic problems”; “I feel able to master similar arithmetic problems”; and “I felt confident about my knowledge about the topic today.” The scale reliability (Cronbach’s α) was 0.863.

### Data Analysis

The hypotheses were tested with a 3 × 2 MANOVA with Drawing (no drawing, D, DQ) and Visual Monitoring (V− and V+) as the independent variables and Linear Overgeneralizations and Performance as the dependent variables. There was homogeneity of variance as assessed with Levene’s test (*p* > 0.05). Significant main effects were further analyzed with *post hoc* Tukey tests. The reported *p*-values for Linear Overgeneralizations and Performance were one-tailed due to our directional expectations. We followed De Bock et al. (2003) procedure to ensure the comparability of the results. This included conducting our analysis with only two of four experimental items. Including all four items in the MANOVA revealed the same results because the effect sizes from the two analyses were very similar.

To analyze Perceived Performance, we conducted a 2 × 2 ANOVA with Drawing (D, DQ) and Visual Monitoring (V− and V+) as factors. Homogeneity of variance was confirmed. Because of the exploratory approach, no assumptions were made about the direction of the effects, and two-tailed *p*-values are reported.

## Results

An overview of the mean scores and standard deviations for all variables in the different experimental conditions is presented in Table 2.

**TABLE 2:** Mean scores (and standard deviations) of all variables in the different experimental conditions.

Variable	CG	DV−	DV+	DQV−	DQV+	Total
Linear Overgeneralizations	0.24	0.39	0.49	0.34	0.36	0.36
	(0.39)	(0.43)	(0.46)	(0.43)	(0.43)	(0.43)
Performance	0.48	0.27	0.31	0.22	0.33	0.32
	(0.46)	(0.39)	(0.44)	(0.39)	(0.39)	(0.42)
Perceived Performance	3.73	3.28	3.92	3.34	3.76	3.60
	(0.82)	(0.78)	(0.75)	(0.88)	(0.83)	(0.85)

### Effect of Drawing Strategy on Linear Overgeneralizations and Performance

In line with our expectations, the drawing strategy increased the number of linear overgeneralizations. Students who applied the drawing strategy with a lower quality (D groups) tended to make more linear overgeneralizations than students who did not use this strategy (CG) (43.5% vs. 24.4%). The MANOVA revealed a marginally significant main effect of Drawing on Linear Overgeneralizations [*F*(2,197) = 1.970, *p* = 0.071; *$\eta_{\text{p}}^{2}$* = 0.020]. *Post hoc* comparisons using the Tukey test indicated significant differences (*p* < 0.05, *d*_Cohen_ = −0.461) between students who used the drawing strategy and the control group, which did not draw.

Further, our expectation that the drawing strategy would have a negative effect on performance was confirmed. Students who applied the drawing strategy with a lower quality (D groups) achieved significantly lower performance scores than students who did not use this strategy (28.6% vs. 47.6%). The MANOVA revealed a significant main effect of Drawing on Performance [*F*(2,197) = 4.323, *p* < 0.05; $\eta_{\text{p}}^{2}$ = 0.043], and *post hoc* comparisons indicated significant differences (*p* < 0.05 *d*_Cohen_ = 0.436) between students who applied the drawing strategy and students who did not.

These findings did not interact with the use of the two test booklets (large- or small-sized scaling factors), which were administered to the D and DQ groups but were not administered to the CG group for economic reasons (large scaling factors only). We will elaborate on this point in the results for the third research question (see section “Effect of Visual Monitoring on Linear Overgeneralizations and Performance”). To ensure comparability, we conducted an additional analysis in which only the groups who received the test version with the large scaling factor were included (CG, DV−, DQV−). The results were similar with even stronger effect sizes (Linear Overgeneralizations: $\eta_{\text{p}}^{2}$ = 0.022; Performance: $\eta_{\text{p}}^{2}$ = 0.069).

### Effect of High-Quality Drawing Strategy on Linear Overgeneralizations and Performance

We were able to partly confirm the hypothesis that the high-quality drawing strategy (DQ) would diminish the negative effect of the drawing strategy. As expected, students who used the high-quality drawing strategy engaged in linear overgeneralizations comparably as often as students who did not use the drawing strategy (CG) (35.0% vs. 24.4%). *Post hoc* Tukey tests confirmed that there were no statistically significant differences between students who used the high-quality drawing strategy and the control group (*p* = 0.198, *d*_Cohen_ = −0.261). However, contrary to our expectations, students who used the high-quality drawing strategy did not show significantly fewer linear overgeneralizations than students who used the drawing strategy with a lower quality (D) (35.0% vs. 38.9%, Tukey tests: *p* = 0.211, *d*_Cohen_ = 0.197).

Further, we expected that students who used the high-quality drawing strategy (DQ) would show the same performance as students who did not use the drawing strategy (CG). Contrary to our expectations, Tukey tests indicated that the mean performance score for the DQ group was significantly lower (*p* < 0.05, *d*_Cohen_ = 0.471) than the score for the CG (27.5% vs. 47.6%). Also the comparison of the two drawing conditions yielded results that went contrary to our expectations: The use of high-quality drawing strategy (DQ) did not lead to a higher performance than a lower quality use of the drawing strategy with a lower quality (D) (27.5% vs. 28.6%; *p* = 0.493, *d*_Cohen_ = 0.027).

### Effect of Visual Monitoring on Linear Overgeneralizations and Performance

We expected that the use of a drawing strategy would not hinder problem solving when used for monitoring purposes, referred to here as visual monitoring. Visual monitoring was operationalized by the smaller-sized scaling factor because we assumed that a smaller scaling factor would make relations between objects in the drawing salient and would therefore inspire visual monitoring.

The results did not confirm our expectations. Students in the visual monitoring group did not differ in the number of linear overgeneralizations from students who could not perform visual monitoring (42.0% vs. 32.5%). There was no significant main effect of Visual Monitoring on Linear Overgeneralizations [*F*(1,197) = 0.698, *p* = 0.202; $\eta_{\text{p}}^{2}$ = 0.004], and there was also no effect of the Visual Monitoring × Drawing interaction on Linear Overgeneralizations [*F*(1,197) = 0.334, *p* = 0.282; $\eta_{\text{p}}^{2}$ = 0.002].

Our expectations were not confirmed for performance either: Visual monitoring did not diminish the negative effect of the drawing strategy on performance (32.0% vs. 32.1%). As was already found for the number of linear overgeneralizations, there was no significant main effect of Visual Monitoring on Performance [*F*(1,197) = 1.312, *p* = 0.127; $\eta_{\text{p}}^{2}$ = 0.007], and there was no effect of the Visual Monitoring × Drawing interaction on Performance [*F*(1,197) = 0.337, *p* = 0.281; $\eta_{\text{p}}^{2}$ = 0.002].

### Effect of Drawing Strategy and Visual Monitoring on Perceived Performance

The results of the ANOVA showed that the quality of the drawing strategy did not affect students’ perceived performance [*F*(1,153) = 0.183, *p* = 0.670; $\eta_{\text{p}}^{2}$ = 0.001]. Students who were given the high-quality drawing strategy (DQ) perceived that their performance was the same as students who were given the lower quality strategy (D) (*M* = 3.54, *SD* = 0.88 vs. *M* = 3.58, *SD* = 0.83).

However, the results revealed a significant effect of visual monitoring on students’ perceived performance [*F*(1,153) = 16.357, *p* < 0.01; $\eta_{\text{p}}^{2}$ = 0.097]. Students in the visual monitoring group perceived a significantly higher performance than their peers who could not easily engage in visual monitoring (*M* = 3.84, *SD* = 0.79 vs. *M* = 3.32, *SD* = 0.83).

Further, no significant effect of the Drawing × Visual Monitoring interaction on Perceived Performance was found [*F*(1,153) = 0.571, *p* = 0.571; $\eta_{\text{p}}^{2}$ = 0.004].

## Discussion

The present study was aimed at replicating De Bock et al. (2003) finding that the drawing strategy hinders students’ ability to solve non-linear geometry problems. We also aimed to elaborate on the potential reasons for this finding by addressing two factors: the quality of the drawing strategy and visual monitoring. Furthermore, we performed an exploratory analysis of students’ perceived performance in order to gather information about students’ awareness of linear overgeneralizations with the hope that this would help us interpret the results of our experimental study.

### Negative Effect of the Drawing Strategy

Our results replicated the previous findings of a negative effect of the drawing strategy on the performance of non-linear geometry problems and confirmed the previous assumption that linear overgeneralizations are a prevalent reason. We found that students who applied the drawing strategy (D groups) made more linear overgeneralizations than students who did not draw. Self-generated drawing seems to guide learners toward mistakenly focusing on the linear relationships depicted in the drawings. However, the effect of the drawing strategy on the number of linear overgeneralizations was smaller than the effect for performance, indicating that applying the drawing strategy may have also resulted in other mistakes, perhaps because of the cognitive cost associated with the externalization process (Zhang, 1997).

Further, the replication of the negative effect of drawing on performance indicates that the findings are stable across different samples. Even the solution scores were very similar to the ones reported by De Bock et al. (2003), with a rate of about 75% for incorrect solutions in the drawing group and 50% in the non-drawing group in both studies.

On a global level, the finding that self-generated drawing hinders students’ ability to solve non-linear geometry problems shows that drawing strategy is not a one-size-fits-all solution and stresses the need to elaborate on the factors that determine beneficial strategy use.

### Quality of Drawing Strategy

Following theoretical considerations about the importance of the quality of self-generated drawing that was confirmed in prior research, we expected that the drawing strategy would hinder students’ ability to solve non-linear geometry problems because it would be applied insufficiently when students solved non-linear problems. Therefore, we increased the quality of the drawing strategy by addressing its key feature by explicitly presenting information that is essential for solving the problem.

The results confirmed the importance of the quality of the drawing strategy. Improving the quality of the drawing strategy diminished the increase in linear overgeneralizations that previously resulted from the drawing strategy. In particular, we found that students who used a high-quality drawing strategy did not differ in the number of linear overgeneralizations they made from students who did not use the drawing strategy, whereas students who applied a lower quality drawing strategy made a larger number of linear overgeneralizations compared with non-drawing students. This finding helps to explain the negative effect of self-generated drawing on solving non-linear geometry problems: Applying the drawing strategy in a high quality way ensures that the area, which is a key element of the problem, will be visible in the drawing. This seems to prevent at least some of the students from being guided by their drawing toward mistakenly focusing on elements of the problem that will interfere with their ability to solve the problem, such as the linear properties of the circumference or the side length. However, more efforts are essential for investigating how we can improve drawing quality so that the drawing strategy can become beneficial.

Contrary to our expectations, we found that improving the quality of the drawing strategy did not diminish the negative effect of self-generated drawing on performance, although it did diminish the negative effect with respect to the number of linear overgeneralizations. Apparently, improving the quality of the drawing strategy did not help students solve the problems. Even the high-quality use of the drawing strategy did not seem to elicit the visual solution strategies that could help students find the right solution. In line with prior research, this finding points out the lack of visual solution strategies, such as calculating the area by paving the figure in order to solve non-linear geometry problems (De Bock et al., 2002b, 2003). Future research should investigate whether training students to use visual solution strategies can lead to the beneficial use of the drawing strategy.

### Visual Monitoring

Another factor that we addressed in order to explain the negative effect of drawing strategy was visual monitoring, the use of the drawing strategy for monitoring purposes. Monitoring has been identified as essential for problem solving (Flavell, 1979) and was found to be important for detecting linear overgeneralizations (De Bock et al., 2002a). For non-linear geometry problems, we assumed that visual monitoring would take place for problems with small scaling factors but would not for large ones because the non-linear relationship of the areas becomes salient while drawing when small scaling factors are used.

However, the findings did not confirm our expectation that visual monitoring diminishes the effect by which self-generated drawing hinders students’ ability to solve non-linear geometry problems. Visual monitoring did not affect the number of linear overgeneralizations or performance. One potential reason for this finding is that students’ tendency to engage in linear overgeneralizations is very strong and difficult to change by engaging in subtle actions such as visual monitoring (De Bock et al., 2007). Visual monitoring may have helped students recognize the non-linear relationship between the areas while drawing, but because students lacked knowledge of how to proceed, they stuck to their use of the linear models they were familiar with to solve the problem. Another reason might be that students did not even notice the non-linear relationship of the areas while drawing because they did not use the drawing strategy for monitoring. Consequently, our assumption that visual monitoring takes place when the drawing strategy is applied to problems with small scaling factors needs to be reconsidered. Previous research has indicated that, in contrast to experts, students use the drawing strategy only infrequently to monitor their solution processes (Stylianou, 2011), so they might not have engaged in visual monitoring even though the non-linear property of the area was made salient while they were drawing. We need more research on how visual monitoring affects the drawing strategy and on how to clarify the mechanisms that can improve visual monitoring in students.

Taken together, our findings confirmed the idea that applying a strategy can have negative effects on students’ performance. The use of a drawing strategy and its effects on solving non-linear problems demonstrates that more efforts are essential for clarifying which factors, apart from fostering linear overgeneralizations, affect the decrease in students’ performance. On a more general level, we argue that there is a need to also further investigate the negative effects of other strategies and identify why some students are misguided when they apply a specific strategy even when this strategy might be helpful for the majority of students. The quality of strategy use seems to be an important factor that should be addressed more often in research on strategies. In addition, research on cognitive factors such as strategic knowledge about drawing (Lingel et al., 2014; Rellensmann et al., 2019) or on emotional and motivational factors such as enjoyment of drawing and the costs of drawing (Uesaka and Manalo, 2017; Schukajlow et al., 2019b) can contribute to clarifying the conditions under which drawing is helpful and when it has a hindering effect.

### Awareness of Linear Overgeneralizations

On the basis of theoretical considerations, we assumed that increasing the quality of the drawing strategy and enhancing visual monitoring would affect students’ awareness of linear overgeneralizations even if it did not affect their performance. Learners may recognize the non-linear property of the problem but might still stick to linear models because they lack the mathematical knowledge necessary to proceed. Indications of whether students were aware of their linear overgeneralizations could be derived from their perceived performance. If students did not notice that drawing guided them incorrectly toward an inadequate use of linear models, they presumably perceived that their performance was higher than the performance of students who were aware that their solution was probably wrong because they made inappropriate linear assumptions.

In order to validate our findings, we conducted an exploratory analysis of students’ perceived performance. Our findings indicated that neither improving the quality of drawing strategy nor enhancing visual monitoring led to a greater awareness of linear overgeneralizations. This finding is in line with prior research that pointed to the intuitive nature of linear misconceptions (Van Dooren et al., 2004, 2008).

It seems that students also encountered other difficulties while solving the problems, ones that did not rely on the non-linear properties of the problem. Students in the group in which visual monitoring was enhanced by using small scaling factors perceived that their performance was even higher than students in the low visual monitoring group who worked on problems with large scaling factors, although the two groups had the same performance scores. The use of small scaling factors probably led to a higher perceived performance because it facilitated the calculations, but it did not lead to a higher performance because the learners made mistakes on the basis of the linear overgeneralizations that they were not aware of. These points indicate that we also need to investigate other difficulties students encounter in solving non-linear geometry problems with the help of a drawing strategy in order to develop a complete picture of the difficulties encountered while solving non-linear problems.

## Strengths and Limitations

We investigated the effect of the drawing strategy for solving non-linear geometry problems by using an experimental design with drawing conditions and a control group that was not instructed to draw. We implemented a treatment check, which showed that students reliably followed the instructions. However, 19 of 41students in the control group spontaneously made drawings. Therefore, we additionally analyzed an adjusted subsample that included only the students in drawing conditions who actually drew and the students in the control group who did not draw. This analysis showed the same results as the previous analyses.

In order to keep the design of the study as simple as possible, the control group worked only on the test version with large scaling factors. As noted in Section “Effect of Drawing Strategy on Linear Overgeneralizations and Performance,” we conducted additional analysis to ensure the comparability of the different drawing conditions. However, the design of our study does not allow to compare students of no drawing and drawing conditions for tests with small scaling factors.

Another important limitation is that our findings are valid for the effects of instructions to make a drawing, but not for spontaneous drawing activity. Descriptive analysis of students’ solutions indicated that students’ spontaneous drawing did not have negative (or even might have slightly positive) effects on students’ achievement-related outcomes. Thus, it might be that spontaneous drawing activity is positively related to students’ achievement-related outcomes. Identifying task- and person-related factors that predict spontaneous use of drawings for non-linear problems is another open question.

A further limitation concerns the operationalization of the factors of drawing quality and visual monitoring. On the basis of theoretical considerations, we assumed that drawing quality would improve if we highlighted the key information given in the problem. Further, we assumed that visual monitoring would be enhanced by the use of small scaling factors compared with large scaling factors. Although both assumptions are plausible, our manipulation might address other factors in addition to these two factors. For example, using small scaling factors decreases the difficulty of the calculations.

As our study was a partial replication of the study by De Bock et al. (2003), we decided to use the same material to render the results as comparable as possible and therefore included only two items in the analyses. As reported in the method section, additional analyses based on all of the four experimental items showed the same results, but future research should increase the number of items to strengthen the validity of these findings.

## Data Availability Statement

The datasets generated for this study are available on request to the corresponding author.

## Ethics Statement

Ethical review and approval was not required for the study on human participants in accordance with the local legislation and institutional requirements. Written informed consent to participate in this study was provided by the participants’ legal guardian/next of kin.

## Author Contributions

SS and JK designed the study. JK analyzed the data and drafted the manuscript. SS revised the manuscript critically for important intellectual content.

## Conflict of Interest

The authors declare that the research was conducted in the absence of any commercial or financial relationships that could be construed as a potential conflict of interest.
